# The stability of flow velocity and intracoronary resistance in the intracoronary electrocardiogram-triggered pressure ratio

**DOI:** 10.1038/s41598-021-93181-0

**Published:** 2021-07-05

**Authors:** Masafumi Nakayama, Nobuhiro Tanaka, Takashi Uchiyama, Takaaki Ohkawauchi, Yusuke Tsuboko, Kiyotaka Iwasaki, Yoshiaki Kawase, Hitoshi Matsuo

**Affiliations:** 1 Department of Cardiovascular Medicine, Gifu Heart Center, 4-14-4 Yabutaminami, Gifu City, Gifu 500‑8384 Japan; 2 Cardiovascular Center, Todachuo General Hospital, Toda, Japan; 3 Department of Cardiology, Hachioji Medical Center, Tokyo Medical University, Hachioji, Japan; 4 College of Humanities and Sciences, Nihon University, Tokyo, Japan; 5 Waseda Research Institute for Science and Engineering, Waseda University, Tokyo, Japan; 6 Department of Modern Mechanical Engineering, School of Creative Science and Engineering, Waseda University, Tokyo, Japan; 7 Department of Integrative Bioscience and Biomedical Engineering, Graduate School of Advanced Science and Engineering, Waseda University, Tokyo, Japan

**Keywords:** Physiology, Circulation, Blood flow

## Abstract

Assessment of coronary artery lesions using the fractional flow reserve and instantaneous flow reserve (iFR) measurements has been found to reduce the incidence of further cardiovascular events. Here, we investigated differences in terms of coronary flow velocity and resistance within the analysis interval between the iFR and the intracoronary electrocardiogram (IC-ECG)-triggered distal/aortic pressure (Pd/Pa) ratio (ICE-T). We enrolled 23 consecutive patients (n = 33 stenoses) who required coronary flow measurements. ICE-T was defined as the average Pd/Pa ratio in the period corresponding to the isoelectric line of the IC-ECG. We compared the index value, flow velocity, and intracoronary resistance during the analysis intervals of the iFR and the ICE-T, both at rest and under hyperemia. ICE-T values and ICE-T intracoronary resistance were both found to be significantly lower, whereas flow velocity was significantly higher than those of the iFR at both rest and under hyperemia (P < 0.001), and all fluctuations in ICE-T values were also significantly smaller than those in the iFR. In conclusion, the ICE-T appears theoretically superior to pressure-dependent indices for analyzing phases with low and stable resistance, without an increase in invasiveness.

## Introduction

Several clinical trials have shown that physiological assessment of coronary artery lesions using fractional flow reserve (FFR) and instantaneous flow reserve (iFR) measurements in coronary interventions contributes to a decrease in cardiovascular events^1–6^. This physiological assessment is necessary to determine the indications for coronary interventions. The iFR value is calculated as the coronary artery distal pressure (Pd) per aortic pressure (Pa) (Pd/Pa) in the absence of hyperemia, during the mid- to end-diastolic or wave-free period (WFP)^7,8^. Although the WFP was originally isolated by applying wave-intensity analysis, the current iFR algorithm (Volcano Corporation, Rancho Cordova, CA, USA; FFR software 2.5) uses an ECG-independent algorithm to identify the diastolic period using the pressure signal only^9^. However, iFR values do not significantly differ from diastolic pressure ratio (dPR)index values analyzed during any diastolic time phase^10^. Previously, we reported that prolongation of the corrected QTU (QTUc) during papaverine-induced hyperemia markedly lowered the iFR values^11^. Those results indicated that the WFP definition, based on aortic pressure, should be discarded. Westerhof et al. argued that the assumption underlying the iFR violates physical principles; however, the iFR and the FFR appear to be associated, indicating the practical utility of the iFR measure^12^.

Intracoronary electrocardiogram (IC-ECG) findings are sensitive and selective in detecting regional myocardial potentials^13–18^. Although the sample size in our previous study was small, we found that, in the period in which the resting Pd/Pa was low, the accuracy of the IC-ECG-triggered resting index (ICE-T) was superior to the iFR in diagnosing myocardial ischemia^19^.

Through minimizing intravascular resistance at maximal hyperemia, a linear relationship can be observed between perfusion pressure and blood flow. In terms of the resting index, minimizing the resistance within the analysis time phase is desirable. We hypothesized that the interval in which the IC-ECG potential is both low and stable might be used as the low-resistance period in coronary artery circulation. However, previous studies have not determined whether intracoronary resistance does indeed decrease during the analysis interval of the ICE-T. This study aimed to investigate the differences between the iFR, the dPR, and the ICE-T in terms of blood flow velocity and resistance within the analysis interval.

## Methods

### Patient selection

In this prospective single-center study, we enrolled 23 consecutive patients who had undergone scheduled coronary angiography and coronary flow reserve measurements for physiological lesion assessments at Todachuo General Hospital between October 2018 and January 2020. All patients had at least one stenosis in a large epicardial artery that required physiological assessment to determine the intervention indications. Exclusion criteria comprised a history of coronary artery bypass surgery, extremely tortuous coronary arteries, acute coronary syndrome, occluded coronary arteries, left main disease, coronary ostial stenosis, congestive heart failure, or an absolute contraindication to the use of adenosine triphosphate (ATP). This study was approved by the Institutional Review Board of Todachuo General Hospital (reference number: 0362), and the study was performed in accordance with the Declaration of Helsinki. Written informed consent was obtained from all participants after a complete explanation of the protocol and potential risks.

### Catheterization and measurement of the instantaneous wave-free ratio at rest and during hyperemia

The coronary flow measurements of coronary artery stenosis were performed in the standard manner. Briefly, a digital archive (ComboMap) with a 0.014-inch dual sensor-equipped guidewire (Combowire; Philips-Volcano, San Diego, CA, USA) was used for all physiological measurements. A bolus of intracoronary nitrates (200–300 μg) was administered to all patients prior to the introduction of the pressure wires. The pressure was calibrated to normal atmospheric pressure prior to inserting the wires and was equalized at the tip of the catheter before advancing the catheter into the distal stenotic lesion.

The Doppler sensor's position was manipulated until an optimal and stable blood velocity signal was obtained distal to the lesion. ATP was then intravenously administered at a rate of 140 μg/kg/min for 3 min until steady-state hyperemia was achieved. Aortic pressure (Pa), coronary pressure (Pd), and flow velocity (V) were continuously recorded at rest and throughout the induction of maximum hyperemia. The IC-ECG was recorded during the physiological measurements by connecting the proximal tip of the Combo wire to the unipolar lead terminal of a multichannel electrocardiogram recorder (RMC-4000 M Cardio Master with EP amplifier system [JB400G]; Nihon Koden, Tokyo, Japan, or AXIOM Sensis HEMO EP128; Siemens AG, Munich, Germany) using a sterile double-alligator connector. These systems allow simultaneous multichannel recordings of ECGs of limb and chest leads during IC-ECG recordings. The IC-ECG data were stored digitally for offline analysis.

### iFR, dPR, and ICE-T analysis

The pressure data were directly extracted from the digital archive of the ComboMap device console. Using the data from the time of pressure equalization, the time phases of the Pd and flow velocity data were synchronized based on the Pa waveform. To identify variations in the pressure parameters during the WFP, the iFR was calculated using the pressure data from three heartbeats included within the automatic iFR calculation data. The iFR was calculated as the Pd/Pa ratio during the WFP (from 25% into diastole until 5 ms prior to the end of diastole)^8^. The diastolic pressure ratio (dPR) was calculated as the Pd/Pa ratio during the entire diastole period. The start of diastole was determined as the nadir of the dicrotic notch on the Pa, and the end of diastole as 50 ms before the upstroke in Pa from the subsequent ventricular contraction.

The IC-ECG data were analyzed using the multichannel ECG recorder as digital data. ECGs were examined by scaling up the sampling speed by 100 mm/s and the ECG signal amplitude by 10 mm/mV. We detected R-wave peaks using the method reported by Lin et al. and we considered the time phase between adjacent R-wave peaks as a single cardiac cycle^20^. The IC-ECG was smoothed by applying a Savitzky-Golay filter^21^. The first peak of the cardiac cycle was determined to be the T wave. Similarly, the last peak was determined to be the P wave. The isoelectric line was considered as the T-P segment preceding the QRS (or QS) complex. The slope of the IC-ECG was < 1 mV/mSec between T and P waves and was labeled as the isoelectric line. When multiple lines were identified as isoelectric lines, only the longest line was automatically detected as the final isoelectric line. The time from the Q point to the start and the end of the isoelectric line was measured.

**Figure 1:**
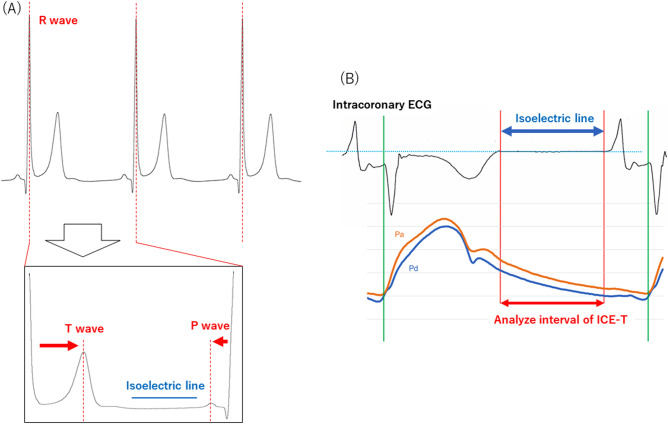
Calculation method for the intracoronary electrocardiogram-based pressure index. (**A**) The R wave was automatically detected and the cardiac cycle was identified. The slope of the intracoronary electrocardiogram, which was < 1 mV/mSec between T and P waves, was labeled as the isoelectric line. (**B**) First, the Q point of the intracoronary electrocardiogram (IC-ECG) was synchronized with the start points of the systolic phase in the pressure waveform. The IC-ECG-triggered distal/aortic pressure (Pd/Pa) ratio was defined as the average of Pd/Pa in the period corresponding to the isoelectric line.

The start points of the systolic phase in the pressure waveform and the Q point in the IC-ECG were regarded as the same point, and the time-phases were synchronized. The IC-ECG-triggered Pd/Pa ratio (ICE-T) value was defined as the average of the Pd/Pa ratio in the period corresponding to the isoelectric line (Fig. 1).

### Data analysis

**Figure 2:**
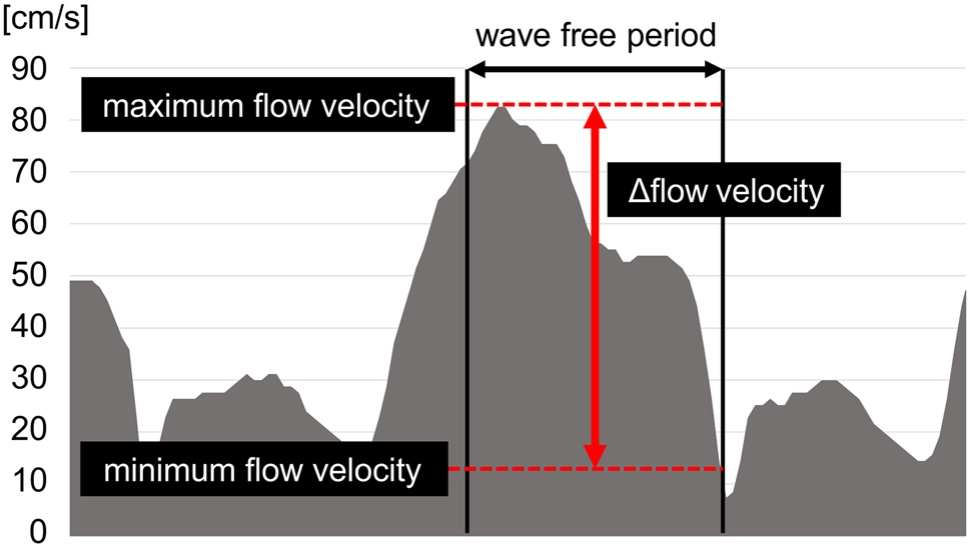
Definition of Δflow velocity. Differences between the minimum and the maximum flow velocity during the analysis interval of each index were defined as Δflow velocity. ΔPd/Pa and ΔResistance were similarly defined. *Pd/Pa* distal/aortic pressure.

The patients’ baseline clinical characteristics, including the number and locations of stenotic lesions, were determined. The index values (iFR, dPR, and ICE-T values) were analyzed offline using MS-Excel (Microsoft Corp., Redmond, WA, USA) software. The intracoronary resistance (R) was calculated as the Pd value divided by the flow velocity at the same point in time. All indices were determined in a fully automated manner for three consecutive heartbeats and were then averaged. The differences between the minimum and maximum flow velocities during the analysis interval of each index were defined as Δflow velocity (ΔV) (Fig. 2). The ΔPd/Pa and ΔR were similarly defined. The index value, mean V, mean R, ΔPd/Pa, ΔV, and ΔR values of the dPR and ICE-T were compared with the iFR at rest and during hyperemia. The periods used for the analysis of the ICE-T and the dPR were also compared. These comparisons were also performed for the left anterior descending coronary artery (LAD) and non-LAD arteries, respectively.

### Statistical analysis

Numerical data were expressed as mean ± standard deviation (SD). Paired *t*-tests were used for comparisons of the pressure parameters, flow velocity, resistance, and analysis period between the iFR, ICE-T, and dPR both at rest and during hyperemia. To examine the internal reliability of the index values, flow velocity, and resistance of each index observed during the three beats, we used an intraclass correlation coefficient (ICC). ICC values < 0.5 indicated poor reliability, values between 0.5 and 0.75 indicated moderate reliability, values between 0.75 and 0.90 indicated good reliability, and values > 0.90 indicated excellent reliability. P-values < 0.05 were considered statistically significant. Statistical analyses were performed using SPSS (SPSS 19; IBM Corporation, Armonk, NY, USA) software.

### Human subjects/informed consent statement

All procedures followed were in accordance with the ethical standards of the responsible committee on human experimentation (institutional and national) and with the Helsinki Declaration of 1975, as revised in 2000. Informed consent was obtained from all patients included in the study.

## Results

### Patient characteristics

Patient clinical characteristics are shown in Table 1. Twenty-three patients participated in this study. Coronary flow reserve and IC-ECG recordings were measured at 33 lesions, with all measurements proving successful and interpretable. The physiological assessment was most often conducted at the LAD coronary artery (51.5%). The patients’ mean age was 68 ± 11 years, and 19 (82.6%) patients were men. Five patients (21.7%) had a history of myocardial infarction.

**Table 1:** Patients’ clinical characteristics.

Number of patients	23
Male, %	19 (82.6%)
Age, years	68 ± 11
Body weight, kg	66 ± 13
Body height, cm	162 ± 7
**Measured artery:**	
Total	33
LAD	17 (51.5%)
LCX	8 (24.2%)
RCA	8 (24.2%)
**Medical history, %:**	
Hypertension	17 (73.9%)
Diabetes mellitus	7 (30.4%)
Dyslipidemia	19 (82.6%)
Current smoking	8 (34.8%)
Prior myocardial infarction	5 (21.7%)

*LAD* left anterior descending artery, *LCX* left circumflex artery, *RCA* right coronary artery.

### A comparison of the index values, pressure parameters, flow velocity, resistance, and the analysis period between the iFR and the ICE-T

Pressure, flow velocity, and resistance data were normally distributed for each group, as assessed using a Shapiro–Wilk test (P > 0.05). The index pressure parameters, resistance, flow velocity, and the analysis period are shown in Table 2. The ICE-T value and resistance of the ICE-T were significantly lower than the iFR value and resistance of the iFR at rest and during hyperemia. The iFR value was significantly lower than the dPR value; however, there was no significant difference in resistance between the ICE-T and the dPR at rest and during hyperemia.The flow velocity of the ICE-T was significantly higher than that of the iFR at rest and during hyperemia. The flow velocity of the iFR was significantly lower than that of the dPR at rest and during hyperemia. The ΔPd/Pa, ΔV, and ΔR of the iFR were significantly larger than those of the ICE-T, and significantly smaller than those of the dPR. The periods used for ICE-T analysis were significantly shorter than those used for the iFR.

**Table 2:** Index pressure parameters, resistance, flow velocity, and the period used for the analysis.

Rest (n = 33)	iFR	DPR	ICE-T	P-value(iFR vs dPR)	P-value (iFR vs ICE-T)
**Index value**	0.916 ± 0.089	0.919 ± 0.082	0.909 ± 0.094	0.015	0.0002
Max	0.957 ± 0.083	0.994 ± 0.046	0.926 ± 0.093	0.0001	< 0.00001
Min	0.888 ± 0.098	0.883 ± 0.096	0.895 ± 0.095	0.0001	0.062
ΔPd/Pa	0.068 ± 0.038	0.110 ± 0.070	0.031 ± 0.021	0.00002	< 0.00001
**Flow velocity, cm/s**					
Mean	22.2 ± 10.4	23.8 ± 11.3	26.8 ± 12.3	< 0.00001	< 0.00001
Max	33.7 ± 16.5	36.4 ± 17.1	31.3 ± 13.4	< 0.00001	0.015
Min	7.8 ± 3.6	7.4 ± 3.4	22.4 ± 11.3	0.003	< 0.00001
ΔV	25.9 ± 15.8	28.9 ± 16.3	8.9 ± 6.0	< 0.00001	< 0.00001
**Intracoronary resistance**					
Mean	5.2 ± 3.8	5.3 ± 4.1	4.5± 3.9	0.405	< 0.00001
Max	12.1 ± 6.6	13.1 ± 7.1	5.8± 5.9	0.002	< 0.00001
Min	3.2 ± 1.9	3.1 ± 1.8	3.4 ± 2.2	0.011	0.149
ΔR	8.9 ± 5.7	10.0 ± 6.4	2.4± 4.8	0.0003	< 0.00001
Analysis interval, msec	393 ± 86	525 ± 114	133 ± 74	< 0.00001	< 0.00001

ATP (n = 33)	iFR	DPR	ICE-T	P-value (iFR vs dPR)	P-value (iFR vs ICE-T)
Index value	0.850 ± 0.099	0.856 ± 0.097	0.832 ± 0.113	0.001	0.00002
Max	0.916 ± 0.076	0.954 ± 0.074	0.857 ± 0.108	< 0.00001	< 0.00001
Min	0.806 ± 0.113	0.803 ± 0.112	0.812 ± 0.115	0.029	0.018
ΔPd/Pa	0.110 ± 0.056	0.151 ± 0.069	0.045 ± 0.025	< 0.00001	< 0.00001
**Flow velocity, cm/s**					
Mean	43.4 ± 19.7	45.2 ± 20.6	54.0 ± 24.5	0.0002	< 0.00001
Max	61.9 ± 27.0	63.8 ± 27.0	60.2 ± 25.9	0.00006	0.067
Min	19.5 ± 12.1	17.9 ± 11.8	47.3 ± 23.1	0.00002	< 0.00001
ΔV	42.4 ± 19.6	45.9 ± 19.7	12.9 ± 6.9	< 0.00001	< 0.00001
**Intracoronary resistance**					
Mean	2.3 ± 2.3	2.4 ± 2.1	1.8 ± 1.3	0.177	0.005
Max	5.0 ± 4.0	6.3 ± 4.8	2.4 ± 3.5	0.005	< 0.00001
Min	1.4 ± 0.7	1.4 ± 0.6	1.4 ± 0.6	0.019	0.003
ΔR	3.7 ± 3.6	4.9 ± 4.4	1.0 ± 3.2	0.004	< 0.00001
Analysis interval, msec	353 ± 68	470 ± 90	106 ± 50	< 0.00001	< 0.00001

*ATP* adenosine triphosphate, *DPR* diastolic pressure ratio, *ICE-T* intracoronary electrocardiogram-triggered resting index, *iFR* instantaneous flow reserve.

The ICC for index values, flow velocity, and resistance of the dPR, iFR, and ICE-T are shown in Table 3. The ICC for index values and flow and resistance of the ICE-T were all > 0.9, which indicated excellent reliability, and was similar to the iFR and the dPR.

**Table 3:** The internal reliability of index values, flow velocity, and resistance concerning the dPR, the iFR, and the ICE-T.

	dPR	iFR	ICE-T
**Rest**			
Index value	0.998	0.998	0.993
Flow	0.995	0.989	0.982
Resistance	0.988	0.975	0.946
**ATP**			
Index value	0.988	0.985	0.981
Flow	0.977	0.968	0.940
Resistance	0.983	0.984	0.945

*ATP* adenosine triphosphate, *DPR* diastolic pressure ratio, *ICE-T* intracoronary electrocardiogram-triggered resting index, *iFR* instantaneous flow reserve.

A comparison of the iFR and the ICE-T in terms of the artery used for measurement (the LAD artery or non-LAD arteries) and condition (rest or ATP-induced hyperemia) is shown in Table 4. The mean flow velocity of the LAD artery within the iFR analysis interval was higher than that of the non-LAD arteries, and the mean resistance of the LAD artery was lower than that of the non-LAD arteries, although these differences were not significant under either resting or hyperemic conditions, except for flow velocity during hyperemia (flow velocity: at rest, P = 0.055; hyperemia, P = 0.002. Resistance: at rest, P = 0.087; hyperemia, P = 0.097). At rest, in both the LAD and non-LAD arteries, the mean index value and mean resistance of the ICE-T were significantly lower than those of the iFR, whereas the mean flow velocity of the ICE-T was significantly higher than that of the iFR, and the analysis interval of the ICE-T was significantly shorter than that of the iFR. ΔPd/Pa, ΔV, and ΔR were significantly smaller in the ICE-T than in the iFR for both the LAD and non-LAD arteries. In both the LAD and non-LAD arteries, there was no significant difference in mean resistance between the dPR and iFR. The mean flow velocity was significantly higher in the iFR than in the dPR in the LAD artery, and conversely, significantly lower in the iFR than in the dPR in the non-LAD arteries. The ΔPd/Pa, ΔV, and ΔR were significantly smaller in the iFR than in the dPR for both the LAD and non-LAD arteries.

**Table 4:** A comparison of iFR and ICE-T according to LAD or non-LAD artery measurements and condition (A: rest, B: hyperemia).

(A) LAD (n = 17)	iFR	DPR	ICE-T	P-value (iFR vs dPR)	P-value (iFR vs ICE-T)
**Index value**	0.909 ± 0.095	0.914 ± 0.088	0.905 ± 0.101	0.014	0.049
Max	0.941 ± 0.089	0.984 ± 0.046	0.919 ± 0.097	0.003	0.0004
Min	0.883 ± 0.108	0.878 ± 0.105	0.892 ± 0.103	0.010	0.180
ΔPd/Pa	0.059 ± 0.035	0.106 ± 0.067	0.027 ± 0.016	0.001	0.001
**Flow velocity, cm/s**					
Mean	27.5 ± 11.0	25.6 ± 10.3	30.5 ± 11.2	< 0.00001	0.00003
Max	38.2 ± 16.6	41.2 ± 16.8	34.5 ± 11.8	0.0002	0.024
Min	9.2 ± 3.9	8.8 ± 3.8	26.0 ± 10.4	0.002	< 0.00001
ΔV	29.0 ± 16.5	32.4 ± 16.6	8.5 ± 4.1	0.00009	0.00004
**Intracoronary resistance**					
Mean	4.1 ± 1.9	4.1 ± 1.8	3.3 ± 1.5	0.399	0.00003
Max	9.8 ± 3.8	10.5 ± 3.6	4.1 ± 2.1	< 0.00001	< 0.00001
Min	2.8 ± 1.5	2.7 ± 1.2	2.9 ± 1.2	0.762	0.762
ΔR	7.0 ± 3.4	7.8 ± 3.3	1.2 ± 1.3	< 0.00001	< 0.00001
Analysis interval, msec	369 ± 74	492 ± 98	121 ± 74	< 0.00001	< 0.00001

Non-LAD (n = 16)	iFR	DPR	ICE-T	P-value (iFR vs dPR)	P-value (iFR vs ICE-T)
Index value	0.923 ± 0.085	0.925 ± 0.078	0.913 ± 0.090	0.347	0.001
Max	0.973 ± 0.077	0.982 ± 0.045	0.933 ± 0.091	0.023	0.0009
Min	0.895 ± 0.089	0.889 ± 0.087	0.899 ± 0.089	0.005	0.059
ΔPd/Pa	0.078 ± 0.039	0.093 ± 0.074	0.034 ± 0.026	0.008	0.0004
**Flow velocity, cm/s**					
Mean	18.7 ± 9.5	19.8 ± 10.4	22.9 ± 12.6	< 0.00001	< 0.00001
Max	29.0 ± 15.5	31.2 ± 16.3	27.9 ± 14.5	0.008	0.340
Min	6.4 ± 2.8	6.0 ± 2.2	18.6 ± 11.2	0.125	0.0003
ΔV	22.6 ± 14.9	25.2 ± 15.7	9.3 ± 7.6	0.002	0.0007
**Intracoronary resistance**					
Mean	6.4 ± 4.9	6.6 ± 5.4	5.8 ± 5.2	0.276	0.004
Max	14.6 ± 8.1	15.8 ± 8.9	7.5 ± 7.9	0.040	0.0002
Min	3.7 ± 2.3	3.6 ± 2.2	4.0 ± 2.9	0.042	0.156
ΔR	11.0 ± 7.0	12.2 ± 8.0	3.5 ± 6.6	0.025	0.0001
Analysis interval, msec	420 ± 92	559 ± 122	145 ± 73	< 0.00001	< 0.00001

(B) LAD (n = 17)	iFR	DPR	ICE-T	P-value (iFR vs dPR)	P-value (iFR vs ICE-T)
**Index value**	0.861 ± 0.105	0.867 ± 0.101	0.85 ± 0.117	0.0007	0.039
Max	0.918 ± 0.079	0.954 ± 0.067	0.875 ± 0.111	0.0003	0.002
Min	0.822 ± 0.118	0.819 ± 0.117	0.832 ± 0.121	0.150	0.036
ΔPd/Pa	0.095 ± 0.055	0.135 ± 0.063	0.043 ± 0.025	0.0001	0.0004
**Flow velocity, cm/s**					
Mean	53.3 ± 18.2	56.0 ± 18.5	67.0 ± 21.4	0.0001	< 0.00001
Max	74.9 ± 24.3	77.3 ± 23.6	73.2 ± 22.9	0.0005	0.270
Min	24.9 ± 13.4	23.3 ± 13.0	60.1 ± 20.4	0.0003	< 0.00001
ΔV	50.0 ± 17.8	54.0 ± 17.7	13.1 ± 7.6	< 0.00001	< 0.00001
**Intracoronary resistance**					
Mean	1.7 ± 0.9	1.7 ± 0.9	1.3 ± 0.6	0.786	0.0005
Max	3.8 ± 2.4	4.4 ± 2.6	1.5 ± 0.7	0.0004	0.0002
Min	1.2 ± 0.6	1.1 ± 0.6	1.2 ± 0.5	0.089	0.249
ΔR	2.7 ± 2.0	3.2 ± 2.2	0.3 ± 0.2	0.0003	0.0002
Analysis interval, msec	342 ± 71	456 ± 95	98 ± 60	< 0.00001	< 0.00001

Non-LAD (n = 16)	iFR	DPR	ICE-T	P-value (iFR vs dPR)	P-value (iFR vs ICE-T)
Index value	0.838 ± 0.095	0.843 ± 0.094	0.813 ± 0.108	0.099	0.00008
Max	0.915 ± 0.076	0.953 ± 0.083	0.838 ± 0.105	0.002	< 0.00001
Min	0.789 ± 0.109	0.786 ± 0.108	0.791 ± 0.109	0.107	0.287
ΔPd/Pa	0.126 ± 0.053	0.167 ± 0.073	0.047 ± 0.026	0.0007	< 0.00001
**Flow velocity, cm/s**					
Mean	32.9 ± 15.6	33.7 ± 16.3	40.3 ± 20.0	0.175	0.00009
Max	48.2 ± 23.1	49.4 ± 23.2	46.3 ± 21.8	0.043	0.134
Min	13.8 ± 7.2	12.1 ± 7.0	33.8 ± 17.7	0.010	0.00001
ΔV	34.4 ± 18.5	37.3 ± 18.5	12.6 ± 6.3	0.001	0.00002
**Intracoronary resistance**					
Mean	3.0 ± 3.1	3.2 ± 2.8	2.3 ± 1.7	0.191	0.059
Max	6.3 ± 4.9	8.3 ± 5.8	3.5 ± 4.9	0.029	< 0.00001
Min	1.6 ± 0.7	1.6 ± 0.7	1.7 ± 0.6	0.098	0.005
ΔR	4.7 ± 4.6	6.7 ± 5.5	1.8 ± 4.6	0.028	< 0.00001
Analysis interval, msec	364 ± 64	485 ± 86	115 ± 36	< 0.00001	< 0.00001

*DPR* diastolic pressure ratio, *ICE-T* intracoronary electrocardiogram-triggered resting index, *iFR* instantaneous flow reserve, *LAD* left anterior descending artery, *non-LAD* left circumflex artery and right coronary artery.

Under hyperemia, in both the LAD and non-LAD arteries, the ICE-T value was significantly lower than the iFR value, the mean flow velocity of the ICE-T was significantly higher than that of the iFR, and the analysis interval of the ICE-T was significantly shorter. While the mean resistance of the ICE-T was lower than that of the iFR, this difference was only significant in the LAD artery (LAD, P = 0.0005; non-LAD, P = 0.059). ΔPd/Pa, ΔV, and ΔR were significantly smaller in the ICE-T than in the iFR for both the LAD and non-LAD arteries. In both the LAD and non-LAD arteries, the mean flow velocity of the dPR was significantly higher than that of the iFR, and there was no significant difference in mean resistance between the dPR and the iFR. The ΔPd/Pa, ΔV, and ΔR were significantly smaller in the iFR than in the dPR for both the LAD and non-LAD arteries.

**Figure 3:**
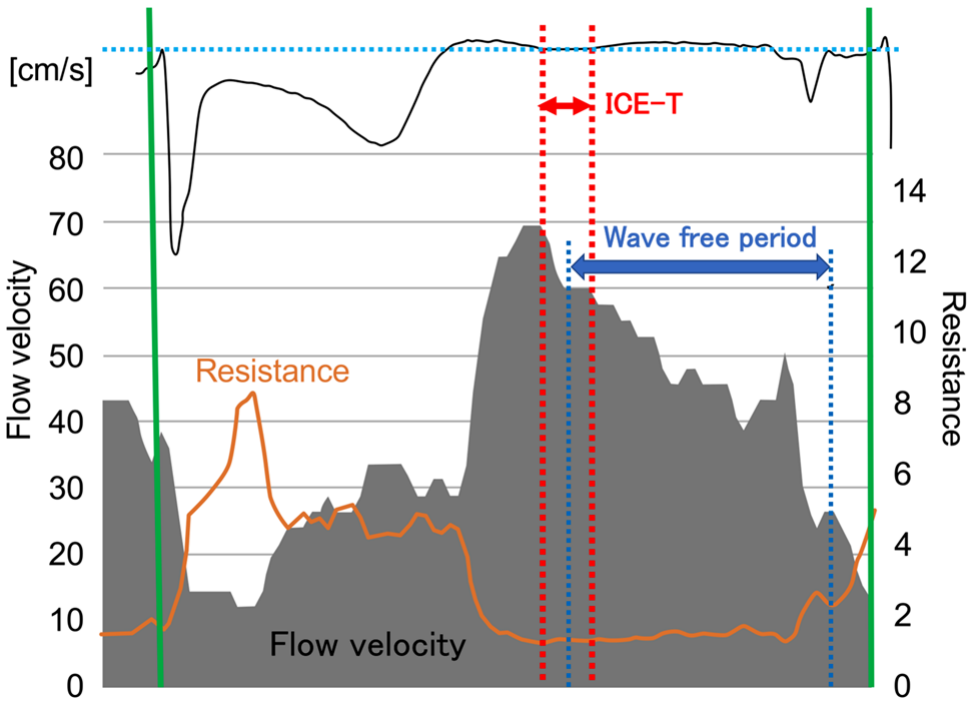
An example of flow velocity, resistance, and intracoronary electrocardiogram recorded in the left anterior descending coronary artery under hyperemia. The flow velocity decreased sharply after reaching a high peak flow. The isoelectric line of the intracoronary electrocardiogram detected the phase immediately after the peak flow velocity. The flow velocity varied greatly during the WFP.

An example of the flow and resistance waveform and the IC-ECG recorded in the LAD artery under ATP-induced hyperemia is shown in Fig. 3. Although the isoelectric phase of the IC-ECG waveform was short, the isoelectric line of the IC-ECG was caught just after the peak flow velocity. The flow velocity varied markedly during the WFP compared with the ICE-T analysis period.

## Discussion

This study showed that determination of the analysis interval using the isoelectric line of the IC-ECG resulted in significantly lower index values, higher flow, and lower resistance than for the iFR. Our findings also indicated that within each analysis interval, all the fluctuations (ΔPd/Pa, ΔV, and ΔR) of the ICE-T were significantly smaller than those of the iFR. The results were the same for the LAD and non-LAD arteries with different blood flow patterns at rest and during hyperemia. Our study findings strongly suggest that the ICE-T, which relies on an IC-ECG, is an ideal index of low intracoronary resistance compared with a pressure-dependent index.

The iFR value is calculated as the Pd/Pa ratio during the mid- to end-diastolic period, which is known as the WFP^7,8^. The diastasis during the diastolic phase is characterized as considerably reduced left ventricular (LV) myocardial activity and fits with the WFP concept. However, both the heart rate and myocardial condition affect the diastasis period to preserve the LV stroke volume^22^. Therefore, even in the same coronary artery of the same patient, the interval of low intracoronary resistance can easily vary. Previously, we reported that prolongation of the QTUc during papaverine-induced hyperemia markedly lowered iFR values^11^. However, the WFP is defined as representing approximately 75% of mid- to late diastole, excluding the initial 25% and the final 5 ms, and neither myocardial activation nor the repolarization time changes the proportion of the WFP in diastole^8,23^. The heart rate has been considered an important factor accounting for the discrepancy between the ischemic diagnosis of the FFR and the iFR because the heart rate only affects the iFR value and not the FFR value^24,25^. Therefore, distinguishing the low intracoronary resistance phase from the aortic pressure might be challenging.

Coronary blood flow predominates in the diastole period^26^. Myocardial contraction compresses the intramyocardial coronary artery, increasing resistance and reducing blood flow during the systolic period. Most of the blood flow in the LAD coronary artery occurs during diastole. However, the right coronary artery has a flow-velocity pattern that is less diastolic-dominant than that of the LAD coronary artery and has different proximal and distal flow-velocity patterns^27–29^. Coronary artery stenosis mainly reduces diastolic blood pressure and coronary blood flow; hence, the diastolic/systolic velocity ratio changes before and after percutaneous coronary intervention^27^. Although many factors affect the blood flow pattern, the iFR is completely dependent on the aortic pressure waveform to determine the analysis interval, namely, the WFP.

Compared with the iFR, the index value of the ICE-T was lower, the flow velocity was higher, and the intracoronary resistance was lower in all conditions where different coronary arteries were measured with or without hyperemia. The ADVISE study reported that the classification agreement (accuracy) between the iFR and the FFR was 94%; however, around the cut-off point for ischemic diagnosis, the iFR-FFR concordance rate was < 50%^30^. Our previous study findings indicated that the ICE-T is better than the iFR in terms of agreement with an ischemic diagnosis according to the FFR, although that study had a small sample size. In particular, the ICE-T had a higher concordance rate with the FFR for the diagnosis of ischemia in borderline cases such as the iFR adenosine zone (0.86 ≤ iFR ≤ 0.93; accuracy, overall; ICE-T 90%, iFR, 72.5%; adenosine zone; iFR 65%, ICE-T 80.0%)^21^. The usefulness of the IC-ECG in predicting microvascular obstruction in myocardial infarction^31^ and post-procedural myocardial injury in angina pectoris has also been reported^32^. These reports indicate that the local myocardial condition can be assessed sensitively using the IC-ECG. The combined use of pressure wire and the IC-ECG to assess myocardial ischemia and myocardial viability simultaneously may reduce the cost of cardiac magnetic resonance imaging and stress myocardial scintigraphy. The ICE-T requires a shorter analysis period than the iFR because it can selectively detect finer electrical potentials representing cardiac muscle activity near the tip of the pressure wire where the pressure sensor is located^33^. Therefore, the ICE-T measure might select an analysis interval with lower intracoronary resistance than the aortic pressure-dependent iFR measure regardless of the measurement conditions.

The ICE-T value under hyperemia was clinically significantly lower than the iFR value (ICE-T 0.832 ± 0.113, iFR 0.850 ± 0.099, P = 0.00002). Although the sample sizes in both studies were small, the diastolic-FFR (d-FFR) has been reported to be useful for diagnosing ischemia and determining blood flow compared with whole-cardiac cycle-FFR^34,35^. However, simultaneous measurement of intracoronary and left ventricular pressure are needed to measure the d-FFR; therefore, it is not widely used because it requires two arterial punctures and is more invasive. The d-FFR is not widely used due to the complexity of its measurement. This study showed that measurement of the ICE-T was simple, accurate, and non-invasive. We consider that determination of the d-FFR in the LAD coronary artery may contribute to an appropriate diagnosis of myocardial ischemia because the greater part of coronary blood flow occurs during the diastolic phase of the cardiac cycle. Although further studies are needed to assess its clinical significance, the hyperemic ICE-T may represent a novel d-FFR index. The widespread use of the ICE-T requires the development of an automated analysis system for IC-ECG.

This study had some limitations. First, the study was conducted on a relatively small number of patients. Second, it is possible that the pressure wire might capture the electrical potential proximal to the stenosis. Nevertheless, we recently reported that the IC-ECG was captured near the tip of the pressure wire^33^. Moreover, this system selectively used the low myocardial electrical activity phase. Therefore, although the analysis interval is short due to potential noise from the proximal coronary arteries, its influence on the index value is likely to be small. Nevertheless, the ICE-T is a promising index for the accurate diagnosis of myocardial ischemia due to coronary artery stenosis. Further multi-center studies are needed to confirm the clinical significance of the ICE-T. In conclusion, the ICE-T, based on the isoelectric line of the IC-ECG, showed significantly lower index values, higher flow, and lower resistance than for the iFR, and all the ICE-T fluctuations were significantly smaller than those of the iFR at rest and during hyperemia. The ICE-T measurement is consistent when selecting low and stable resistance phases in contrast to using a pressure-dependent index. The ICE-T and iFR values differed significantly but involved only small differences. At rest, the pressure gradient at the stenosis is small because the blood flow velocity through the stenosis is slow. However, the clinical significance of small index value differences between ICE-T and iFR values remains unknown. Therefore, further studies are needed to confirm the clinical significance of the new ICE-T index at rest and under hyperemia.
